# Can neurocognition, brain neurotrophic factor, triglyceride, and total cholesterol predict suicidal ideation in first‐episode Han Chinese patients with schizophrenia?

**DOI:** 10.1002/brb3.3499

**Published:** 2024-04-29

**Authors:** Li Dongxia, Ma Li, Feng Yingying

**Affiliations:** ^1^ Wuhan Mental Health Center Wuhan Psychological Hospital Wuhan City China

**Keywords:** brain neurotrophic factor, neurocognition, schizophrenia, suicidal ideation

## Abstract

**Objective:**

Previous studies have suggested that the suicide rate of patients with schizophrenia is high. This study investigates factors influencing suicidal ideation in first‐episode schizophrenia patients, focusing on cognitive function, brain‐derived neurotrophic factor (BDNF), triglyceride (TG), and total cholesterol (TC) in patients with first‐episode schizophrenia.

**Methods:**

A total of 123 patients with first‐episode schizophrenia and 38 healthy controls were included in the study. The patients were divided into suicidal and nonsuicidal ideation groups based on the Beck Scale for Suicidal Ideation, and they were assessed with Positive and Negative Syndrome Scale (PANSS). Cognitive function was assessed using the Chinese version of the MATRICS consensus cognitive battery (MCCB) and the serum BDNF, TG, and TC were detected. The main statistical methods include *t*‐test, χ^2^ test, multivariate logistic regression analysis, receiver operating characteristic (ROC) curve analysis, and the DeLong test.

**Results:**

26.02% of patients exhibited suicidal ideation. Higher PANSS and TC levels were risk factors, while higher MCCB scores and BDNF levels were protective factors. ROC analysis indicated AUCs of 0.630, 0.724, and 0.762 for serum BDNF, PANSS, and MCCB, respectively, with a combined AUC of 0.870.

**Conclusion:**

Serum BDNF level, PANSS score, and MCCB score can be used as auxiliary predictors of suicidal ideation in schizophrenic patients. Combining these three indicators can effectively predict suicidal ideation in schizophrenic patients.

## Graphical abstract

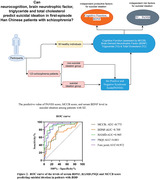

## 1 INTRODUCTION

Schizophrenia is a serious mental disorder that imposes a severe economic burden on patients, their caregivers, and the society, with a worldwide lifetime prevalence of 0.48% (Simeone et al., 2015) and 0.83% (Chan et al., 2015) in China. Suicide is a serious global health challenge (World Health Organization, 2019). Studies have found that patients with schizophrenia have a higher risk of suicide than the normal population, and suicide was one of the important causes of premature death in schizophrenia (Gohar et al., 2023; Plana‐Ripoll et al., 2019). Suicidal ideation is the main link and inevitable stage of the psychological process that leads to suicidal behavior (Melle et al., 2017). Suicidal ideation is also strongly associated with suicide (Chapman et al., 2015). A meta‐analysis of 26 studies covering 5079 patients with schizophrenia showed that the lifetime and time‐point prevalence of suicidal ideation were 34.5% and 29.9%, respectively (Bai et al., 2021). Research on factors influencing suicidal ideation in patients with schizophrenia is of great importance for reducing suicidal behavior and protecting patient safety.

Schizophrenia has a heterogeneous genetic and neurobiological background that affects early brain development, and clinical symptoms can be classified as positive, such as hallucinations and delusions, and negative, such as apathy, poor thinking, and cognitive symptoms (Kahn et al., 2015). Recent studies have shown that cognitive symptoms are the most important predictor of social function in patients with schizophrenia. This is because cognitive deficits may hinder these patients’ optimal adaptive abilities, thereby imposing neurocognitive limitations on social functional outcomes (Kuo et al., 2018). Additionally, prolonged untreated schizophrenia is associated with selective cognitive decline. Both neurodegeneration and neurodevelopmental dysfunction may contribute to this cognitive impairment (Stone et al., 2020). It has been suggested that cognitive impairment in patients with schizophrenia may be related to suicide. For example, Yin et al. (2020) found that patients with first‐episode schizophrenia who were more impaired in specific areas of neurocognitive function had a higher probability of suicide attempts. However, there are differing views. For example, Delaney et al. (2012) found that patients who had attempted suicide had better cognitive function than those who had not attempted suicide. A study by Dai et al. (2021) found no significant association between suicide attempts and cognitive function in people with schizophrenia. Studies have found that first‐episode psychosis (FEP) patients already have impaired cognitive function, global cognitive functioning (GCF) is the most reliable predictor of suicidal behavior and severe depressive symptoms, comprehensive neurocognitive assessment may be useful for risk assessment (Canal‐Rivero et al., 2018). The heterogeneity of the sample may have contributed to these different findings.

The literature documents associations between suicide risk, demographic characteristics, and clinical symptoms of schizophrenia, including female sex, marital status, more severe depressive symptoms (Zhong et al., 2019), childhood trauma (Mohammadzadeh et al., 2019), cognitive function, and stigmatization (Stip et al., 2017). However, in addition to the above‐mentioned factors, growing evidence supports the important role of biochemical markers in predicting suicide risk in schizophrenia. Studies have shown that neurotrophins are crucial for the developmental process and plasticity of synapses, and are involved in the development of schizophrenia (Nieto et al., 2021). Plasma brain‐derived neurotrophic factor (BDNF) level may be a potential biomarker of cognitive recovery in acute schizophrenia (Zhang et al., 2018). Furthermore, studies have shown that BDNF can cross the blood‐brain barrier, and BDNF levels measured in peripheral blood may reflect changes in BDNF levels in the brain (Pillai et al., 2010).

Metabolic abnormalities from various causes are associated with an increased risk of cardiovascular disease in patients with schizophrenia, contributing to death (Zhang et al., 2017) and may also be associated with suicide risk. Additionally, studies have shown that dyslipidemia is involved in the development of schizophrenia (Dickens et al., 2021). Rybakowski (2014) revealed significant associations between suicidal ideation and lower levels of total cholesterol (TC), low‐density lipoprotein (LDL), triglyceride (TG), and total lipids in schizophrenia. A systematic review and meta‐analysis also found that suicide attempts are associated with low total cholesterol levels in patients with schizophrenia spectrum disorders (Sankaranarayanan et al., 2021). However, Misiak et al. (2015) found that higher total levels were associated with suicidal ideation in first‐episode female patients with schizophrenia. Another study also showed a positive correlation between serum triglyceride levels and suicidal ideation severity (Fang et al., 2019).

Previous studies have often neglected to consider these factors as a unified whole. As a result, this study strives to comprehensively examine the role of multiple factors, including BDNF, TG, and TC levels, clinical symptoms, and cognitive function, in predicting suicidal ideation among schizophrenic patients. This holistic approach aims to offer a more precise and comprehensive scientific foundation for preventing suicidal behavior in schizophrenic patients.

## 2 MATERIALS AND METHODS

### 2.1 Participants

This is a cross‐sectional study focusing on 123 patients with first‐episode schizophrenia who received treatment at the Wuhan Mental Health Center from February 2022 to February 2023, including 65 males and 58 females. The inclusion criteria for this study were as follows: (a) meeting the International Classification of Diseases, 10th Revision (ICD‐10) criteria for the diagnosis of schizophrenia; (b) age 18−60 years; (c) first episode of schizophrenia and no use of antipsychotics, mood stabilizers, or other psychiatric drugs; and (d) patients and family members providing informed and signed consent. The exclusion criteria were as follows: (a) pregnant and lactating women; (b) comorbid depression, bipolar disorder, and other psychiatric disorders; (c) combined severe cardiocerebrovascular and metabolic diseases and other somatic diseases; and (d) comorbid psychoactive substance abuse. In the same period, 38 healthy individuals with physical examination were selected as the control group, 22 males and 16 females. The inclusion criteria were as follows: (a) age 18−60 years; (b) there are no previous or current mental disorders that meet the ICD‐10 diagnostic criteria; (c) no severe cardiocerebrovascular and metabolic diseases and other physical diseases; (d) no psychoactive substance abuse; (e) not pregnant, lactating women and the latter. This study was approved by the Medical Ethics Committee (Ethical Number: KY201908.7).

### 2.2 Methods

#### 2.2.1 Collection of clinical data

Information on the gender, age, marital status (unmarried, married, separated/widowed), years of education, and occupation was collected from each first‐episode schizophrenia patient. Patients were assessed for psychiatric symptoms using the Positive and Negative Syndrome Scale (PANSS) (Kay et al., 1987), which contains 30 items divided into three subscales: positive symptoms, negative symptoms, and general psychopathology, with higher scores indicating more severe symptoms.

#### 2.2.2 Biochemical measurements

Fasting venous blood was collected from patients with schizophrenia in the early morning after enrollment and from controls at the physical examination visit, centrifuged at 2500 R/min for 20 min, and the upper serum was stored in a −80°C freezer. Serum BDNF levels were measured using sandwich enzyme‐linked immunosorbent assay (ELISA) with dual antibodies (Wuhan Ruixin Bio, Cat. No. rx104552h), and LDL, high‐density lipoprotein (HDL), TG, and TC levels were measured a Beckman au480 automatic biochemical analyzer (USA).

#### 2.2.3 Assessment of cognitive function

Cognitive function was assessed using the Chinese version of the MATRICS Consensus Cognitive Battery (MCCB) (Zou et al., 2009). This assessment was conducted in the morning after the enrollment of patients with schizophrenia, and on the day of the physical examination for healthy controls. The battery comprised nine tests that evaluated seven cognitive domains: speed of processing, attention and vigilance, verbal learning, working memory, problem‐solving, visual learning, and social cognition (Nuechterlein et al., 2004). The Chinese version of the MCCB has undergone co‐norming and standardization in China, demonstrating sufficient clinical validity and reliability in both controls and patients with schizophrenia (Shi et al., 2015).

#### 2.2.4 Assessment of suicidal ideation

The Beck Suicidal Ideation Inventory (Beck et al., 1979) was administered in the morning after enrollment to assess the most recent 1 week or when the past was most depressed or suicidal. Articles 4 (how much you would like your initiative to attempt suicide) and 5 (how much you would like your external strength to end your life) judged the presence or absence of suicidal ideation; any one entry that answered “ weak” or “moderate intensity” considered the presence of suicidal ideation, and no suicidal ideation was considered if any of the two above entries returned “None.” Accordingly, the patients were divided into those with suicidal ideation (*n* = 32) and those without suicidal ideation (*n* = 91).

All assessments for the research subjects are generally completed within two days.

### 2.3 Statistical analysis

The Shapiro–Wilk test was used to perform normality tests. Continuous variable data conforming to the normal distribution were expressed as mean ± standard deviation and analyzed using analysis of variance (ANOVA); continuous variable and ordinal variable data not conforming to the normal distribution were expressed as median (25th percentile to 75th percentile) and analyzed using the Mann–Whitney *U* test. Categorical variables were analyzed using the chi‐square test. In addition, a multivariate logistic regression analysis was conducted to investigate the influencing factors of suicidal ideation in schizophrenia patients, and the predictive value of serum TG, TC, BDNF levels, PANSS scores, and MCCB scores for suicidal ideation in schizophrenia patients was analyzed. Receiver operating characteristic (ROC) curves were plotted. The areas under the curve (AUCs) for each metric were compared using the DeLong test, and the results were corrected using Bonferroni's method. All statistical analyses were performed using SPSS 25.0 software, and a *p*‐value < .05 was considered statistically significant.

## 3 RESULTS

### 3.1 Comparison of the demographics and levels of TG, TC, LDL, HDL, BDNF, and MCCB score between SZ group and the normal control group

There was no significant difference in demographic data, the serum TC, LDL, and HDL level between the two groups, but the serum TG level in the SZ group was higher than that in the normal control group, while the serum BDNF level and MCCB score were lower than those in the normal control group (*p* <.05) (see Table 1).

**TABLE 1:** Comparison of the demographics and levels of TG, TC, LDL, HDL, BDNF, and MCCB score between SZ group and the normal control group.

	SZ group (*n* = 123)	normal control group (*n* = 38)	*t*	*p*
Sex (male/female)	65/58	22/16	0.298	.585
Age (year, $\bar{x}$ * *± *s*)	36.72 ± 8.18	37.35 ± 5.53	−0.540	.591
Marital status (*n*)			0.114	.944
Unmarried	48	14		
Married	61	19		
Divorce/widow	14	5		
Years of education (year, $\bar{x}$ * *± *s*)	12.61 ± 1.43	12.90 ± 1.32	−1.142	.255
TG (mmol/L, $\bar{x}$ * *± *s*)	1.75 ± 0.13	1.51 ± 0.12	10.040	<.001
TC (mmol/L, $\bar{x}$ * *± *s*)	4.22 ± 1.08	3.96 ± 1.12	1.302	.195
LDL (mmol/L, $\bar{x}$ * *± *s*)	3.10 ± 0.95	3.12 ± 0.62	−0.094	.926
HDL (mmol/L, $\bar{x}$ * *± *s*)	1.41 ± 0.43	1.47 ± 0.63	−0.537	.594
BDNF (ng/mL, $\bar{x}$ * *± *s*)	9.57 ± 3.31	12.27 ± 4.17	−3.644	.001
MCCB (score, $\bar{x}$ * *± *s*)	53.19 ± 8.91	58.22 ± 7.74	−3.133	.002

### 3.2 Univariate analysis of suicidal ideation among the SZ

Among the enrolled SZ patients with, 32 patients had suicidal ideation, with a prevalence of suicidal ideation was 26.02% (32/123). Serum BDNF level and MCCB score of the suicidal ideation group were lower than the nonsuicidal ideation group, the proportions of males, PANSS scores, serum TC level were higher than the nonsuicidal ideation group (*p* <.05) (see Table 2).

**TABLE 2:** Univariate analysis of suicidal ideation among the SZ.

	With SI (*n* = 32)	Without SI (*n* = 91)	*x* ^2^/*t*/*U*	*p*
Sex (male/female)	23/9	42/49	6.285	.012
Age (year, $\bar{x}$ * *± *s*)	37.55 ± 7.65	36.43 ± 8.38	0.665	.507
Marital status [*n* (%)]			0.774	.679
Unmarried	12 (25.00)	36 (75.00)		
Married	15 (24.59)	46 (75.41)		
Divorce/widow	5 (35.71)	9 (64.29)		
Years of education (year, $\bar{x}$ ± *s*)	12.67 ± 1.99	12.58 ± 1.18	0.245	.808
TG (mmol/L, $\bar{x}$ * *± *s*)	1.74 ± 0.10	1.75 ± 0.14	−0.460	.647
TC (mmol/L, $\bar{x}$ * *± *s*)	4.75 ± 1.10	4.03 ± 1.01	3.387	.001
LDL (mmol/L, $\bar{x}$ * *± *s*)	2.95 ± 0.67	3.16 ± 1.03	−1.260	.211
HDL (mmol/L, $\bar{x}$ * *± *s*)	1.38 ± 0.30	1.42 ± 0.47	−0.597	.552
PANSS score (score, $\bar{x}$ * *± *s*)	96.67 ± 8,86	89.15 ± 8.38	4.303	<.001
BDNF (ng/mL, $\bar{x}$ * *± *s*)	8.28 ± 3.50	10.03 ± 3.14	−2.627	.010
MCCB (score, $\bar{x}$ * *± *s*)	46.85 ± 8.79	55.42 ± 7.85	−5.150	<.001

### 3.3 Multivariate logistic regression analysis of suicidal ideation among the SZ

Sex, PANSS score, MCCB score, serum BDNF, and TC levels were used as independent variables, and with and without suicidal ideation as dependent variables (0 if absent, 1 if present). Multivariate logistic regression analysis showed that an increased PANSS score was an independent risk factor for suicidal ideation (*p* < .05), and an increased MCCB score and serum BDNF levels were independent protective factors against suicidal ideation (*p* < .05) (see Table 3).

**TABLE 3:** Multivariate logistic regression analysis of suicidal ideation among the SZ.

	β	SE	Wald *x* ^2^	*p*	OR	95％ CI
Sex	−0.881	0.552	2.548	.110	0.414	0.140–1.223
BDNF	−0.228	0.083	7.479	.006	0.796	0.676–0.937
TC	0.129	0.317	0.167	.683	1.138	0.612–2.116
PANSS score	0.114	0.041	7.780	.005	1.121	1.034–1.214
MCCB score	−0.128	0.034	14.263	<.001	0.880	0.823–0.940

### 3.4 The predictive value of PANSS score, MCCB score, and serum BDNF level in suicidal ideation among patients with SZ

**FIGURE 1:**
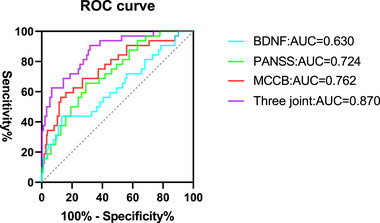
ROC curve of the levels of serum BDNF, MCCB, and PANSS scores predicting suicidal ideation in patients with SZ.

ROC curve analysis showed that the areas under the curve (AUC) of the PANSS score, MCCB score, and serum BDNF level alone and in combination to predict suicidal ideation in patients with SZ were 0.724, 0.762, 0.630, and 0.870, respectively. The AUC of the three combined indices for predicting suicidal ideation in patients with SZ was greater than that of each index alone. The DeLong test showed that the three combined indices were more effective than the serum BDNF, PANSS, and MCCB scores in predicting suicidal ideation in patients with schizophrenia

(see Table 4 and Figure 1).

**TABLE 4:** Comparison of individual and combined predictive efficacy of serum BDNF, MCCB, and PANSS scores on suicidal ideation in patients with SZ.

Indexes	AUC	Predictive efficacy (individual vs. combined)	95％ CI	Cut‐off	Sensitivity(％)	Specificity(％)	Youden index
BDNF	0.630	<0.0001^a^	0.512–0.749	7.03 ng/mL	43.75	86.81	0.306
PANSS	0.724	0.0015^a^	0.627–0.822	93.74 scores	65.63	71.43	0.371
MCCB	0.762	0.0158^a^	0.662–0.862	46.79 scores	56.25	86.81	0.431
Combined	0.870		0.799–0.940	–	90.63	68.13	0.588

^a^ Passed the Bonferroni's correction.

## 4 DISCUSSION

Schizophrenia is a psychotic syndrome characterized by symptoms of hallucinations, delusions, and disorganized speech, as well as impaired cognition, including executive and memory functions (Marder & Cannon, 2019). It is a serious mental disorder with a high suicide rate, with a lifetime prevalence of suicidal ideation of 34.5% (95% CI: 28.2–40.9%) in patients with schizophrenia (Bai et al., 2021). Suicidal ideation has been reported to be the most powerful predictor of suicide attempt (Fang et al., 2018). Among the studies found, patients with schizophrenia and suicidal ideation had a 5.8‐fold higher risk of suicide than those without (Hubers et al., 2018). In the present study, the incidence of concomitant suicidal ideation in 123 patients with schizophrenia was 26.02%, which was similar to the 25.8% (95% CI: 14.7–41.1%) reported by Dong et al. (2019) through a meta‐analysis. Another study on Chinese patients with schizophrenia found a lifetime prevalence of suicidal ideation of 19.22% (Liang et al., 2023), indicating a higher incidence of suicidal ideation in patients with schizophrenia.

Neurotrophins are a family of protein molecules innervated by nerves that are essential for neuronal growth and survival. They can enter the nerve terminal via receptor‐mediated endocytosis and promote the synthesis of proteins somatically to maintain neuronal growth and development; when deficient, they affect neuronal growth and development, leading to the development of schizophrenia symptoms (Man et al., 2018; Nieto et al., 2021). BDNF is involved in the regulation of neurodevelopment, neuroprotection, synaptic plasticity, learning and memory, and studies have shown that decreased BDNF levels are associated with impaired cognition in schizophrenia (Lu et al., 2014; Yang et al., 2019). The present study's results revealed lower BDNF levels in patients with schizophrenia compared to healthy controls, aligning with the findings reported by Man et al. (2018) and Shoshina et al. (2021). Furthermore, the study demonstrated that elevated serum BDNF levels served as an independent protective factor against suicidal ideation in schizophrenia patients; the reason for this may be that BDNF has a trophic effect, and elevated BDNF can promote nerve growth and development, repair damaged nerves, reduce schizophrenia symptoms, and reduce suicidal ideation. In addition, it has been found that the presence of the Met allele in the Val66Met polymorphism of the BDNF gene may be associated with the risk of attempted suicide in patients with schizophrenia (Bolat Kaya et al., 2022).

The results of the present study show that an increased PANSS total score is an independent risk factor for suicidal ideation in patients with schizophrenia. The clinical symptoms of schizophrenia can be classified into positive symptoms such as hallucinations and delusions and negative symptoms such as anhedonia, apathy, and cognitive symptoms (Kahn et al., 2015). Positive and negative symptoms and cognitive dysfunction are core features of schizophrenia. Previous studies have found an association between clinical symptoms and suicidal ideation in patients with schizophrenia; however, the results have been inconsistent. The results of a systematic review suggest that blunted affect may play an important role in the development of suicidal behavior in patients with schizophrenia; however, it may hinder the identification of suicide risk because we did not observe appreciable distress or hopelessness, whereas positive symptoms such as hallucinations and delusions were more likely to be detected by physicians because of their clinical manifestations in the acute phase of the illness (Grigoriou & Upthegrove, 2020). Bornheimer (2016) found that depressive and positive symptoms (particularly hallucinations and delusions) of the PANSS were independently predictive of suicidal ideation in schizophrenia. Yin et al. (2023) showed that the presence of auditory hallucinations was closely related to patients' current suicidal ideation or behavior. Additionally, a meta‐analysis conducted by Cassidy et al. (2018) indicated that patients with schizophrenia and suicidal ideation had higher PANSS total scores and depressive symptoms. Liu et al. (2023) also found general psychopathological symptoms of the PANSS to be an independent risk factor for suicidal ideation in adult Chinese patients with schizophrenia. A study conducted in middle‐aged and elderly Chinese patients with schizophrenia showed that female sex, higher PANSS positive scores, higher depression scores, and higher story recall scores in cognitive functioning were independently associated with suicide attempts (Huang et al., 2021).

Cognitive dysfunction is a core feature of schizophrenia. Studies have found cognitive impairment throughout the disease course in patients with schizophrenia (Sheffield et al., 2018), and it is the main determinant of the impairment of the patient's social function (Santesteban‐Echarri et al., 2017). It may be important to assess whether suicidal ideation is associated with cognitive function, which may have implications for suicide prevention. The results of the present study showed that an increased MCCB total score was an independent protective factor against suicidal ideation in patients with schizophrenia. This is consistent with the findings of Yin et al. (2020), who found that hospitalized patients with schizophrenia with suicidal ideation had significantly lower scores in all seven neurocognitive domains of the MCCB than those without suicidal ideation, and that suicidal ideation was mainly associated with lower scores in working memory and processing speed. However, these findings are inconsistent. Fang et al. (2019) showed that higher cognitive function, especially visuospatial skills, may increase the risk of suicidal ideation in schizophrenia. Villa et al. (2018) also suggested that stronger cognitive abilities, especially verbal learning and self‐reflection, may be independent risk factors for suicidal ideation and behavior.

We established the values of three indices, serum BDNF, PANSS score, and MCCB score, for predicting suicidal ideation in patients with schizophrenia using ROC analysis. The area under the curve (AUC) of BDNF was 0.630, with a 95% confidence level (0.512–0.749), with a sensitivity of 43.75% and a specificity of 86.81%, AUC of PANSS score was 0.724, with a 95% confidence level (0.627–0.822), with a sensitivity of 65.63% and a specificity of 71.43%, AUC of MCCB score was 0.762, with a 95% confidence level (0.662–0.862), with a sensitivity of 56.25% and a specificity of 86.81%. The AUC of the combined prediction of serum BDNF, PANSS score, and MCCB score was 0.870, which was higher than the AUC of each factor alone, its Youden index was 0.588, which was higher than the Youden index predicted by each factor alone. The DeLong test showed that the three combined indices were more effective than the serum BDNF, PANSS, and MCCB scores in predicting suicidal ideation in patients with schizophrenia.

The strengths of this study are that all patients with schizophrenia were first episode‐free of confounders, such as long‐term medication, and the MCCB used to measure cognitive function is also an international standard measurement tool. However, the sample size of this study was small, and the enrolled patients were all inpatients; however, our current findings have important clinical implications. The combined prediction of serum BDNF, PANSS, and MCCB scores is expected to improve the accuracy of predicting suicidal ideation in patients with schizophrenia. Follow‐up studies need further validation of the findings in multicenter case studies with large samples and further analysis of the mechanisms by which the indicators' involvement in this study affects cognitive function and suicidal ideation in patients with schizophrenia.

## AUTHOR CONTRIBUTIONS

**Li Dongxia**: Funding acquisition; writing—original draft. **Ma Li**: Methodology; data curation. **Feng Yingying**: Funding acquisition; writing—review and editing.

## CONFLICT OF INTEREST STATEMENT

The authors report no competing interests.

### ETHICS STATEMENT

The trial was examined and authorized by the hospital Ethics Committees.

### PATIENT CONSENT STATEMENT

Prior to participation, written informed consent was obtained from all participants.

### PEER REVIEW

The peer review history for this article is available at https://publons.com/publon/10.1002/brb3.3499.

## Data Availability

The data that support the findings of this study are available from the corresponding author upon reasonable request.
